# Annexin5 Plays a Vital Role in *Arabidopsis* Pollen Development via Ca^2+^-Dependent Membrane Trafficking

**DOI:** 10.1371/journal.pone.0102407

**Published:** 2014-07-14

**Authors:** Jingen Zhu, Xiaorong Wu, Shunjie Yuan, Dong Qian, Qiong Nan, Lizhe An, Yun Xiang

**Affiliations:** MOE Key Laboratory of Cell Activities and Stress Adaptations, School of Life Sciences, Lanzhou University, Lanzhou, China; University of Delhi South Campus, India

## Abstract

The regulation of pollen development and pollen tube growth is a complicated biological process that is crucial for sexual reproduction in flowering plants. Annexins are widely distributed from protists to higher eukaryotes and play multiple roles in numerous cellular events by acting as a putative “linker” between Ca^2+^ signaling, the actin cytoskeleton and the membrane, which are required for pollen development and pollen tube growth. Our recent report suggested that downregulation of the function of *Arabidopsis* annexin 5 (Ann5) in transgenic Ann5-RNAi lines caused severely sterile pollen grains. However, little is known about the underlying mechanisms of the function of Ann5 in pollen. This study demonstrated that Ann5 associates with phospholipid membrane and this association is stimulated by Ca^2+^ in vitro. Brefeldin A (BFA) interferes with endomembrane trafficking and inhibits pollen germination and pollen tube growth. Both pollen germination and pollen tube growth of *Ann5*-overexpressing plants showed increased resistance to BFA treatment, and this effect was regulated by calcium. Overexpression of *Ann5* promoted Ca^2+^-dependent cytoplasmic streaming in pollen tubes in vivo in response to BFA. Lactrunculin (LatB) significantly prohibited pollen germination and tube growth by binding with high affinity to monomeric actin and preferentially targeting dynamic actin filament arrays and preventing actin polymerization. Overexpression of *Ann5* did not affect pollen germination or pollen tube growth in response to LatB compared with wild-type, although Ann5 interacts with actin filaments in a manner similar to some animal annexins. In addition, the sterile pollen phenotype could be only partially rescued by Ann5 mutants at Ca^2+^-binding sites when compared to the complete recovery by wild-type Ann5. These data demonstrated that Ann5 is involved in pollen development, germination and pollen tube growth through the promotion of endomembrane trafficking modulated by calcium. Our results provide reliable molecular mechanisms that underlie the function of Ann5 in pollen.

## Introduction

Pollen germination and pollen tube growth are key steps in the double fertilization sexual reproduction process of flowering plants [1]. Many signaling molecules and cellular events, including Ca^2+^, pH, phosphatidyl inositol, endocytosis, exocytosis, vesicle trafficking, plasma membrane fusion and actin filament reorganization, have been shown to be separately or synergistically involved in regulating the accurate polarized growth of the pollen tube [2].

The pollen tube is a typical cell in the apex that exhibits rapid, polarized growth. During the polarized growth of pollen tubes, the components of the cell wall and plasma membrane must be delivered to the tip of the pollen tube by precise vesicle trafficking and then secreted to enable membrane fusion [3]. Thus, highly ordered cytoplasmic streaming and membrane fusion are critical factors in polarized pollen tube growth. In addition, the actin cytoskeleton and Ca^2+^ are well known to have important influences on pollen tube growth, and a disorder in either leads to cessation of pollen tube growth [4]–[7]. Ca^2+^ also plays a crucial role in determining the velocity of cytoplasmic streaming, membrane fusion and actin cytoskeleton reorganization [8]–[13]. Some recent studies have shown that the actin cytoskeleton in the pollen tube not only functions as a track for vesicle trafficking but also directs the distribution of vesicles [6]. If this actin cytoskeleton was destroyed, vesicles would not be accurately transported to the specific locus of the membrane at the apex, causing the pollen tubes to stop growing [14]. Furthermore, previous reports have indicated that the interaction between actin and the plasma membrane initiates membrane fusion [15]. Consequently, the actin cytoskeleton and Ca^2+^ are indispensable for many membrane-related physiological activities and may synergistically regulate these activities [16]. However, little is known about the underlying mechanisms that regulate pollen development and pollen tube growth via the coordinated regulation of the membrane, actin cytoskeleton and Ca^2+^ because the functional “linkers“ between these factors remain unknown.

The annexin family is a class of proteins that can bind to the membrane in a Ca^2+^-dependent manner. The members of this family share an evolutionarily conserved structure that can be found in a wide variety of eukaryotic cells. In mammalian cells, annexins have been shown to be involved in crucial cellular processes, such as vesicle trafficking, membrane organization, signal transduction, actin cytoskeletal dynamics and ion exchange [17]–[20]. Annexins from plants and animals have highly similar protein function and structure, with core domains composed of four homologous repeats of approximately 60–70 amino acids that contain a conserved Ca^2+^- and membrane-binding motif. In recent years, annexins from many different plants have been isolated, and their functions have been studied. As with annexins in animals, those in plants have the conserved function of the protein family and participate in many significant physiological activities, such as the cell cycle [21], pollen and seed germination [22], [23], [77], tuber development of cassava [24], cotton fiber elongation [25], petunia petal senescence [26], strawberry fruit ripening and gall ontogeny [27], primary root growth and lateral root formation [28]–[30], vascular development [31] and cork formation [32]. Most of these events are linked to Ca^2+^ signaling and membrane function. In addition, certain plant annexins can also function in environmental stimuli. There are eight annexin genes with deduced amino acid sequence identities varying from 29% to 83% in the *Arabidopsis* genome (*AnnAt1*–*8*) [33]. AnnAt1 and AnnAt4 participate in osmotic stress and ABA signaling in a Ca^2+^-dependent manner, which could implicate them in exocytosis or cell cycle control [34], [35]. Some abiotic stress stimuli, such as light, gravity, phosphate starvation, metal stress, cold, drought and oxidation, can alter the expression and abundance of plant annexins [36]–[38]. Interestingly, Blachbourn et al. demonstrated the presence of lily annexins at the apex of the pollen tube via an immunofluorescence assay, and these annexins also bound to vesicles in the pollen tube in a Ca^2+^-dependent manner [39]. These results suggest that annexin may function as an important “linker“ between the membrane, actin cytoskeleton and Ca^2+^ in the polarized growth of the pollen tube. However, this hypothesis has not been conclusively demonstrated in the research on the involvement of annexin family proteins in the polarized growth of pollen tubes.

Many reports have shown that membrane-related processes, such as membrane trafficking, fusion and exocytosis, are highly active throughout pollen development and necessary for normal pollen development. It has been demonstrated that membrane trafficking and deformation by the ER and Golgi occur as early as the uninucleate late microspore stage. After the first mitotic division, a large vacuole is split into smaller vacuoles by invaginations of the tonoplast at several points from one side to the opposite side of the vacuole. The small vacuoles then disperse throughout the cytoplasm, and some fuse with the plasma membrane, which becomes intensely convoluted. The observation that many membrane-bound structures appeared between the convoluted plasma membrane and the intine implicated that exocytosis seems to occur in pollen [40],[41]. Our previous data demonstrated that *Ann5* is predominantly expressed in pollen after the bicellular stage and pollen tube formation. In addition, downregulation of the function of Ann5 in transgenic RNAi lines led to severely sterile pollen grains at the bicellular stage of pollen development [42]. However, the mechanisms underlying the function of Ann5 in pollen remain largely unclear.

In this study, biochemical analyses indicated that Ann5 binds to phospholipid membrane and this association is stimulated by Ca^2+^. Moreover, Ann5 interacts with actin filaments in vitro. The pollen germination, pollen tube growth and cytoplasmic streaming of plants overexpressing *Ann5* were more resistant to BFA treatment in a Ca^2+^-regulated manner in vivo. The defects observed in pollen grains of the Ann5UTR-RNAi lines could be only partially recovered by Ann5 mutants at Ca^2+^-binding sites, which further indicated that the Ann5 Ca^2+^-dependent phospholipid-binding activity is essential for pollen development.

## Results

### Ann5 Binds to Phospholipid Membranes in a Ca^2+^-dependent Manner

**Figure 1:**
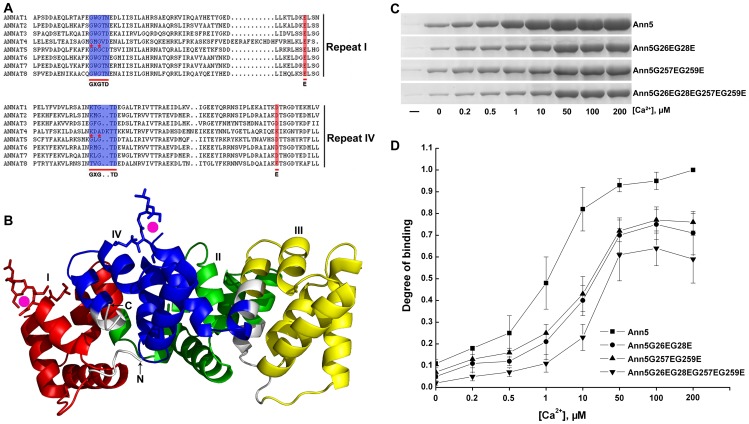
Ann5 binds to negatively charged phospholipids and this association is stimulated by Ca^2+^. (A) Multiple sequence alignment of the deduced amino acid sequences of repeats I and IV from *Arabidopsis thaliana* AnnAt1 to 8. Ca^2+^-binding sites of type II GXGTD-(37 residues)-E/D are indicated by blue and red shadows. The residues with red asterisks indicate the locations of PCR-based site-directed mutagenesis. The mutants of repeats I and IV were named Ann5G26EG28E and Ann5G257EG259E, respectively, and the conserved glycine residues were replaced by glutamic acid residues. (B) Predicted three-dimensional structure of the Ann5 protein. The annexin core domain was composed of four homologous repeats that are colored in red (repeat I), green (repeat II), yellow (repeat III) and blue (repeat IV) and shaped as a slightly curved disc. The convex surface on which calcium ions bind (magenta spheres) participates in peripheral membrane binding. The type II Ca^2+^-binding sites are labeled with sticks, and the ribbon illustrates the highly α-helical structure. (C) Phospholipid-binding properties of the recombinant His6-Ann5, His6-Ann5G26EG28E, His6-Ann5G257EG259E and His6-Ann5G26EG28EG257EG259E proteins. The individual protein (50 µg) was incubated with liposomes (1∶1 PC/PS) in the presence of increasing Ca^2+^ concentrations. (-) denotes that the reaction mixtures contained neither liposomes nor Ca^2+^. (D) Comparison of the Ca^2+^-dependent phospholipid binding abilities of Ann5 and its mutants using densitometry analysis of the signal intensities of blots as described in (C). The phospholipid binding amount of Ann5 in 200 µM Ca^2+^ is normalized as 1 (control). The relative intensities are displayed as fold-binding over the control. Values represent mean ± _SD_ (n = 6).

Annexins from plants and animals have highly similar protein structure [18], [37], [38], [78]. Using a structural mimetic of homology modeling, Ann5 was shown to have a unique structure, with a characteristic tetrad structure of homologous internal repeats at the carboxy-terminal end and a variable amino-terminal region that allows Ann5 to associate with membranes and Ca^2+^ in a peripheral and reversible manner, thus providing a link between Ca^2+^ signaling and membrane functions. The Ann5 core domain, which is composed of four homologous repeats of approximately 70 amino acids, contains the conserved Ca^2+^- and membrane-binding motif, which is shaped as a slightly curved disc. The convex surface participates in peripheral membrane binding. Calcium ions also bind to the convex side of the core and face the membrane when Ann5 binds to phospholipids. Type II Ca^2+^-binding sites within the first and fourth repeats exhibit higher affinities for calcium and consist of the conserved sequence GXGTD-(37 residues)-E/D (Figure 1A) [18], [43]. The concave side of the disc faces the cytoplasm and is thus available for regulatory binding or interactions with other components within the cytosol in a Ca^2+^-regulated manner. A Ca^2+^ ion bridge between Ann5 and phospholipids facilitates the docking of Ann5 onto membranes [38], [44] (Figure 1B, Figures S1A and S1B).

Calcium-dependent binding to phospholipid membranes is a key characteristic of annexins. Thus, the Ca^2+^-dependent phospholipid-binding activity of Ann5 was tested using a co-sedimentation experiment. Recombinant Ann5 and two Ann5 mutants in which the conserved glycine residue was replaced by a glutamic acid residue in repeats I and IV, namely, Ann5G26EG28E and Ann5G257EG259E, were expressed in *E. coli* and purified (Figure S1C). Subsequently, preformed liposomes composed of 1∶1 Phosphatidyl cholines/Phosphatidyl serines (PC/PS) were incubated with equal amounts of Ann5 or its mutants at various concentrations of Ca^2+^ (0, 0.2, 0.5, 1, 10, 50, 100 and 200 µM) at neutral pH and then centrifuged (Figure S1D). GST and AtAnn1 were used as the negative and positive controls, respectively (Figures S1E and S1F). As shown in Figures 1C and D, Ann5, Ann5G26EG28E and Ann5G257EG259E bound to and co-sedimented with the liposomes in a Ca^2+^-dependent manner. However, Ann5G26EG28E and Ann5G257EG259E exhibited a significant reduction in liposome binding in the presence of various concentrations of Ca^2+^ when compared with Ann5. Furthermore, the quadruple mutant, Ann5G26EG28EG257EG259E, showed a marked decrease in liposome binding in comparison to Ann5G26EG28E or Ann5G257EG259E, demonstrating that the site-directed mutagenesis of glycine residues diminished the phospholipid binding activity of Ann5. Interestingly, small amounts of Ann5, Ann5G26EG28E and Ann5G257EG259E could even bind to the liposomes in the absence of calcium, and this binding manner might serve as a platform for Ann5's Ca^2+^-dependent binding (Figure 1C). These results indicate that Ann5 binds to phospholipid membranes and this association is stimulated by Ca^2+^ in vitro.

### Overexpression of *Ann5* Conferred Resistance to BFA during Pollen Germination and Tube Growth

**Figure 2:**
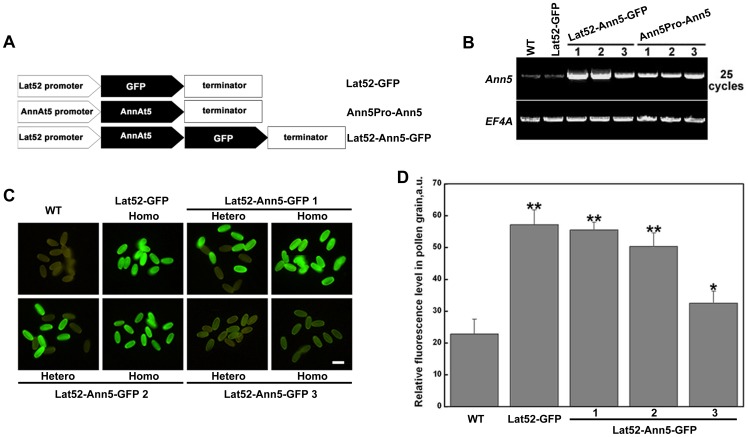
Identification of the constructs concerning Ann5. (A) Schematic representations of the constructs concerning Ann5. (B) *Ann5*-specific primers were designed to identify the level of overexpression of the *Ann5* transcript by RT-PCR performed for 25 cycles. Total RNA was extracted from open flowers of the wild-type (WT), Lat52-GFP, Lat52-Ann5-GFP 1, 2 and 3 and Ann5Pro-Ann5 1, 2 and 3 homozygous lines. *EF4A* was used as the control. (C) Pollen grains from the WT, Lat52-GFP and Lat52-Ann5-GFP 1, 2 and 3 lines were observed by epi-fluorescence microscopy with a GFP filter. Homo, homozygous lines; Hetero, heterozygous lines. Half of the pollen grains in the heterozygous lines expressed Ann5-GFP, and the other half did not express Ann5-GFP. Bar  = 20 µm. (D) Densitometry analysis of the results presented in (C). Fluorescence intensities (arbitrary units) across the whole pollen grain were calculated. More than 50 pollen grains for each line were quantified. Values represent the means ± _SD_. *P<0.05 and **P<0.01 by Student's *t* test.

*Ann5* is abundant in mature pollen grains and elongating pollen tubes; however, it was difficult to investigate the function of *Ann5* during pollen germination and pollen tube growth using the RNAi lines, as *Ann5* suppression resulted in the abortion of pollen grains before the mature pollen stage [42]. Thus, to further understand the in vivo function of *Ann5*, *Ann5* ectopic overexpression lines were generated by expressing Lat52-Ann5-GFP or Ann5Pro-Ann5 constructs in a wild-type background. The Lat52-GFP construct was used as the control (Figure 2A). RT-PCR analysis indicated that the Lat52-Ann5-GFP and Ann5Pro-Ann5 significantly upregulated the expression of the *Ann5* gene to different levels in pollen grains compared with the wild-type and Lat52-GFP lines. Furthermore, the increase in the *Ann5* transcript level in the Lat52-Ann5-GFP 1 and 2 lines was much higher than that in the Lat52-Ann5-GFP 3 and Ann5Pro-Ann5 1, 2 and 3 lines (Figure 2B). Additionally, the fluorescence levels observed under the same microscope setting in the Lat52-Ann5-GFP 1 and 2 pollen grains were significantly higher than that in the Lat52-Ann5-GFP 3 pollen grains (Figures 2C and 2D). Representative Lat52-GFP, Lat52-Ann5-GFP 1 and 3 and Ann5Pro-Ann5 1 homozygous lines were chosen for further analyses.

**Figure 3:**
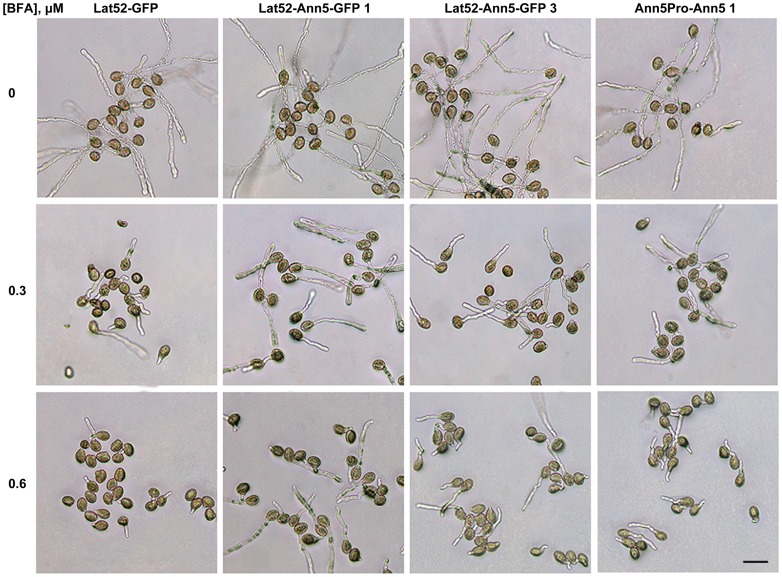
Overexpression of *Ann5* increased the resistance of pollen germination and tube growth to BFA. Representative micrographs of pollen grains and tubes from the *Ann5*-overexpressing lines growing on *Arabidopsis* pollen germination medium containing 0.3 µM or 0.6 µM BFA for 3 h in vitro. Medium containing 50 nM DMSO was used as the control. Bar  = 50 µm.

Initially, we examined whether overexpression of *Ann5* affected pollen germination and tube growth. Surprisingly, there were no distinct or significant morphological differences (i.e., in size and shape) in pollen germination or pollen tube elongation between the wild-type and *Ann5*-overexpressing plants (Figure 3). Considering that Ann5 binds to negatively charged phospholipids in a Ca^2+^-dependent manner in vitro, we next sought to determine whether the pollen germination and pollen tube growth of *Ann5*-overexpressing lines responded to BFA treatment differently. BFA interferes with endomembrane trafficking and strongly inhibits pollen germination and pollen tube growth in a dose-dependent manner through the inability to recruit COPI coat proteins onto Golgi membranes and to block ER to Golgi transport [45]–[47]. The germination rate of pollen grains from *Ann5*-overexpressing lines was analyzed by incubation in *Arabidopsis* pollen germination medium containing 0.3 µM or 0.6 µM BFA for 3 h in vitro. The pollen germination rates in response to 0.3 µM BFA were 51.7%, 85.6%, 68.5% and 66.7% for the Lat52-GFP, Lat52-Ann5-GFP 1, Lat52-Ann5-GFP 3 and Ann5Pro-Ann5 1 lines, respectively (P<0.01 for the Lat52-Ann5-GFP 1 line, and P<0.05 for the Lat52-Ann5-GFP 3 and Ann5Pro-Ann5 1 lines). Additionally, with the addition of 0.6 µM BFA, the average pollen germination rate dropped to 6.8%, 53.6%, 23.3% and 22.1% for the Lat52-GFP, Lat52-Ann5-GFP 1, Lat52-Ann5-GFP 3 and Ann5Pro-Ann5 1 lines, respectively (P<0.01). The pollen germination rates between the Lat52-GFP and *Ann5*-overexpressing plants were significantly different. The Lat52-Ann5-GFP 1 line showed a higher germination rate than the Lat52-Ann5-GFP 3 and Ann5Pro-Ann5 1 lines, which was correlated with the level of overexpression of ectopic *Ann5* (Figures 2 and 3, Table 1). Furthermore, a similar tendency was found for the pollen tube growth of the *Ann5*-overexpressing lines in response to BFA treatment (Table 1). Taken together, these results demonstrated that pollen germination and pollen tube growth of *Ann5*-overexpressing plants significantly increased the resistance to BFA treatment compared with the wild-type, which implied that *Ann5* was involved in endomembrane trafficking and most likely promoted processes in response to BFA stimulation via binding to negatively charged phospholipids.

**Table 1:** Quantitative analysis of pollen germination rates and pollen tube lengths of the Lat52-GFP, Lat52-Ann5-GFP 1 and 3, and Ann5Pro-Ann5 1 homozygous lines.

	BFA (µM)	Lat52-GFP	Lat52-Ann5-GFP 1	Lat52-Ann5-GFP 2	Ann5Pro-Ann5 1
Pollen germination rate, %	0	89.7±1.5	89.4±2.3	92.6±1.1	91.8±1.6
	0.3	51.4±3.1	85.3±3.2**	68.7±3.2*	66.4±2.3*
	0.6	6.9±1.7	53.8±4.5**	23.3±3.3**	22.1±2.5**
Average pollen tube length, µm	0	361.7±10.4	353.3±16.8	352.8±9.7	349.0±10.1
	0.3	231.4±9.1	304.8±8.6**	271.5±9.3*	267.9±9.6*
	0.6	192.4±7.0	262.9±8.6**	235.2±7.5*	226.7±6.7*

For pollen germination rate, the pollen germinated for 3 h in the presence of 0.3 or 0.6 µM BFA. Eight hundred pollen grains and tubes were counted. For pollen tube length, the pollen germinated normally for 2 h and was then treated with 0.3 or 0.6 µM BFA for 2 h. A total of 500 pollen tubes were measured. Values represent the means ± _SD_. *P<0.05 and **P<0.01 by Student's *t* test.

### *Ann5* Overexpression Did Not Influence Pollen Germination and Tube Growth in Response to LatB

**Figure 4:**
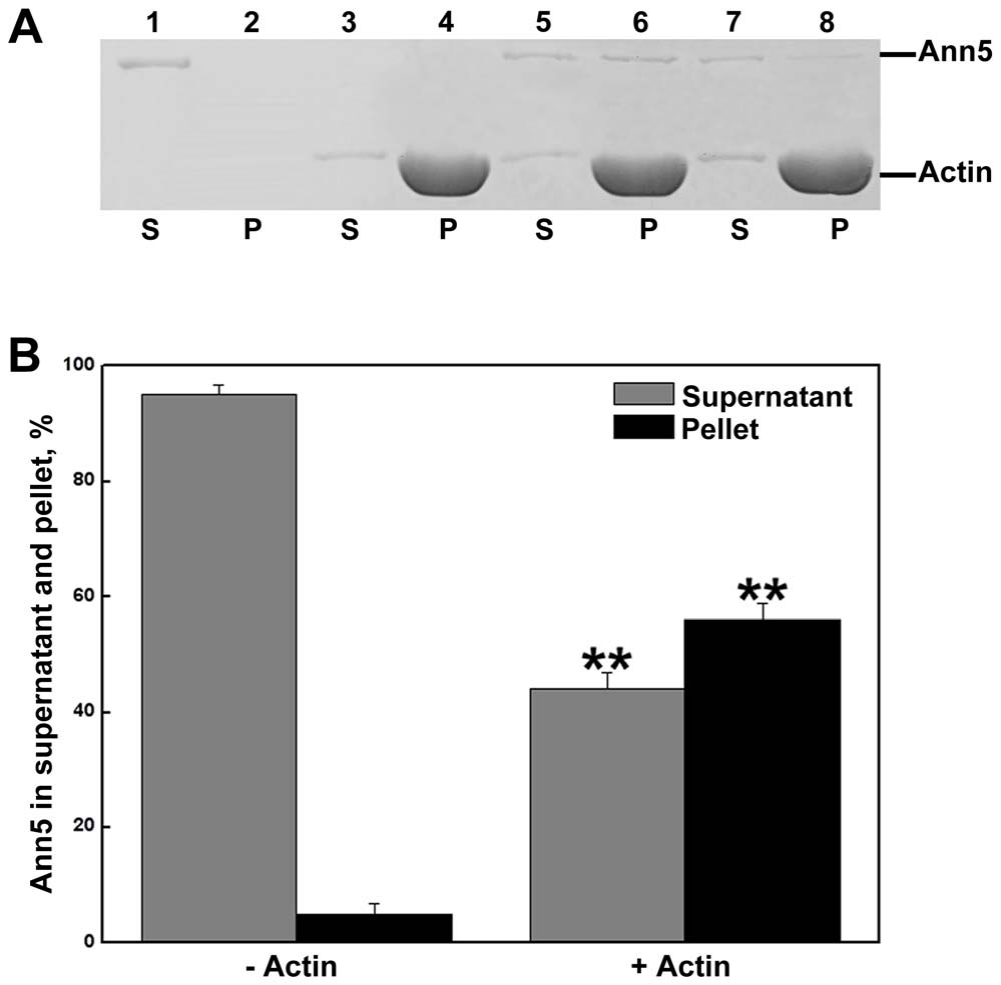
Ann5 binds to actin filaments, as revealed by a high-speed co-sedimentation assay. (A) Either 0.6 µM (lanes 5 and 6) or 0.3 µM (lanes 7 and 8) Ann5 was incubated with 4 µM F-actin at 22°C for 1 h and then centrifuged at 100,000 g for 1 h. Equal amounts of the supernatant (S) and pellet (P) were separated by SDS-PAGE and stained with Coomassie Brilliant Blue R. Samples in lanes 1 and 2 represent the supernatant and pellet of 0.6 µM Ann5 alone. Lanes 3 and 4 contain 4 µM F-actin alone. (B) The percentages of Ann5 in the supernatant and pellet were determined in the absence (lane 1 and 2) or presence (lane 5 and 6) of F-actin and are presented in (A) by densitometry. Values represent the means ± _SD_ (n = 9). **P<0.01 by Student's *t* test.

Many annexins throughout the animal and plant kingdoms have been described as actin-binding proteins [18]. A high-speed co-sedimentation assay was performed to investigate the actin-binding activity of Ann5. GST and GST-AtCROLIN1 were used as the negative and positive controls, respectively (Figure S2) [83]. As shown in Figures 4A and B, the amount of sedimentable Ann5 increased in a dose-dependent manner in the presence of polymerized actin, and, when alone, most Ann5 was found in the supernatant as a soluble protein, suggesting that recombinant Ann5 bound to actin filaments in vitro.

**Figure 5:**
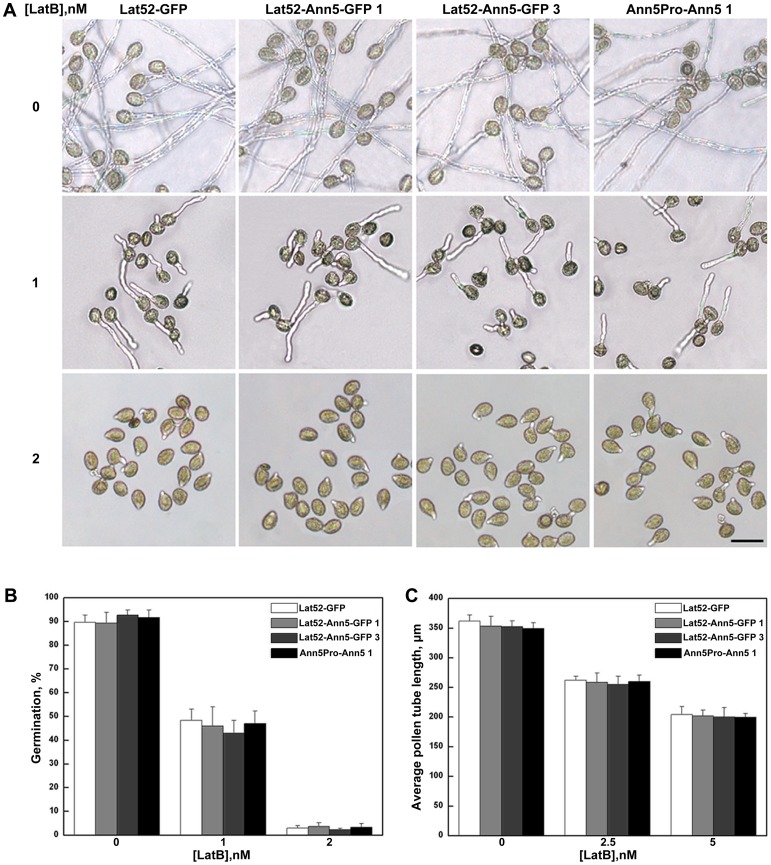
*Ann5* overexpression did not influence pollen germination and tube growth in response to LatB. (A) Representative images of pollen grains and tubes from the Lat52-GFP, Lat52-Ann5-GFP 1 and 3 and Ann5Pro-Ann5 1 homozygous lines growing on germination medium containing 1 or 2 nM LatB for 3 h. Bar  = 50 µm. (B) Pollen germination ratios of the Lat52-GFP, Lat52-Ann5-GFP 1 and 3 and Ann5Pro-Ann5 1 homozygous lines after the pollen had germinated for 3 h in the presence of 1 or 2 nM LatB. Values represent the means ± _SD_. Eight hundred pollen grains and tubes were counted. (C) Pollen tube lengths of the Lat52-GFP, Lat52-Ann5-GFP 1 and 3 and Ann5Pro-Ann5 1 homozygous lines after the pollen germinated normally for 2 h and then for 2 h in the presence of 2.5 or 5 nM LatB. Values represent the means ± _SD_. Five hundred pollen tubes were measured.

We next sought to verify whether the pollen germination and tube growth of the *Ann5*-overexpressing lines responded to the actin depolymerization reagent latrunculin B (LatB). However, unlike BFA treatment, pollen germination and pollen tube growth of the *Ann5*-overexpressing lines were the same as in the control lines when treated by LatB (Figures 5A to C). Although the functional significance of the annexin–actin interaction is poorly understood, *Ann5* has been postulated to be involved in intracellular trafficking events, and actin filaments serve as molecular tracks in pollen grains and tubes [36], [48], [49]. We speculated that if we used LatB to destroy the F-actin organization in the cell, the transport routes that Ann5 depended on would be disrupted, which would disrupt pollen germination and tube growth in the *Ann5*-overexpressing lines similar to that in the wild-type.

### The Effects of *Ann5* on Pollen Germination and Tube Growth Are Regulated by Its Ca^2+^-dependent Membrane Binding Activity

**Figure 6:**
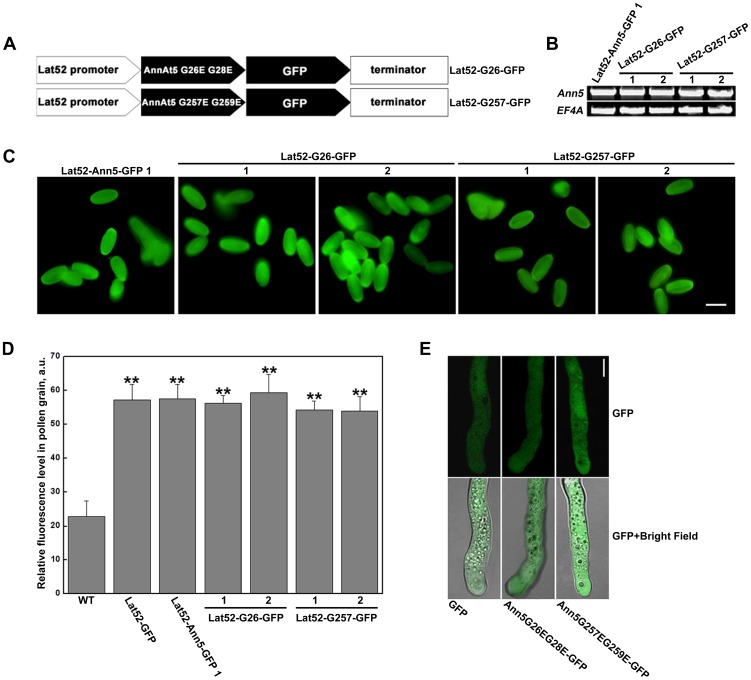
Identification of the *Ann5G26EG28E*- and *Ann5G257EG259E*-overexpression constructs. (A) Scheme of the domain structures of the Lat52-G26-GFP and Lat52-G257-GFP-overexpression constructs. Ann5G26EG28E and Ann5G257EG259E were abbreviated to G26 and G257, respectively. (B) RT-PCR was performed to examine the transcript levels of *Ann5*, *G26* and *G257* using *Ann5*-specific primers. Total RNA was isolated from open flowers of the Lat52-Ann5-GFP 1, Lat52-G26-GFP 1 and 2 and Lat52-G257-GFP 1 and 2 homozygous lines. *EF4A* was used as the control. (C) Representative micrographs of GFP fluorescence in pollen grains from the Lat52-Ann5-GFP 1, Lat52-G26-GFP 1 and 2 and Lat52-G257-GFP 1 and 2 homozygous lines. Bar  = 20 µm. (D) Quantitative analysis of the fluorescence intensities (arbitrary units) across the whole pollen grain from the WT, Lat52-GFP, Lat52-Ann5-GFP 1, Lat52-G26-GFP 1 and 2, and Lat52-G257-GFP 1 and 2 homozygous lines. Thirty pollen grains were quantified for each line. Values represent the means ± _SD_. **P<0.01 by Student's *t* test. (E) Subcellular localization of Ann5G26EG28E and Ann5G257EG259E in pollen tubes. GFP alone was used as the control. Confocal laser scanning microscopy images of single planes in the pollen tube center were obtained. Bars  = 5 µm.

The addition of Ca^2+^ enhanced the phospholipid binding activity of Ann5 to PC/PS in vitro, and, thus, the function of Ann5 was most likely regulated by Ca^2+^ fluctuations within pollen cells. To obtain a more direct indication of whether Ca^2+^ affected the function of Ann5 in endomembrane trafficking in vivo, the pollen germination and pollen tube growth in the *Ann5G26EG28E-* and *Ann5G257EG259E*-overexpressing lines (named Lat52-G26-GFP and Lat52-G257-GFP) were tested following BFA treatment (Figure 6A). Pollen from the Lat52-Ann5-GFP 1 plant was used as the control. Using RT-PCR and fluorescence intensity analyses, representative overexpression lines undergoing similar levels of transcription of *Ann5* were chosen for further analysis (Figures 6B to D). Both Ann5G26EG28E and Ann5G257EG259E were evenly distributed in the cytosol of the pollen tube, which was similar to that of Ann5-GFP or GFP alone (Figure 6E) [42].

**Figure 7:**
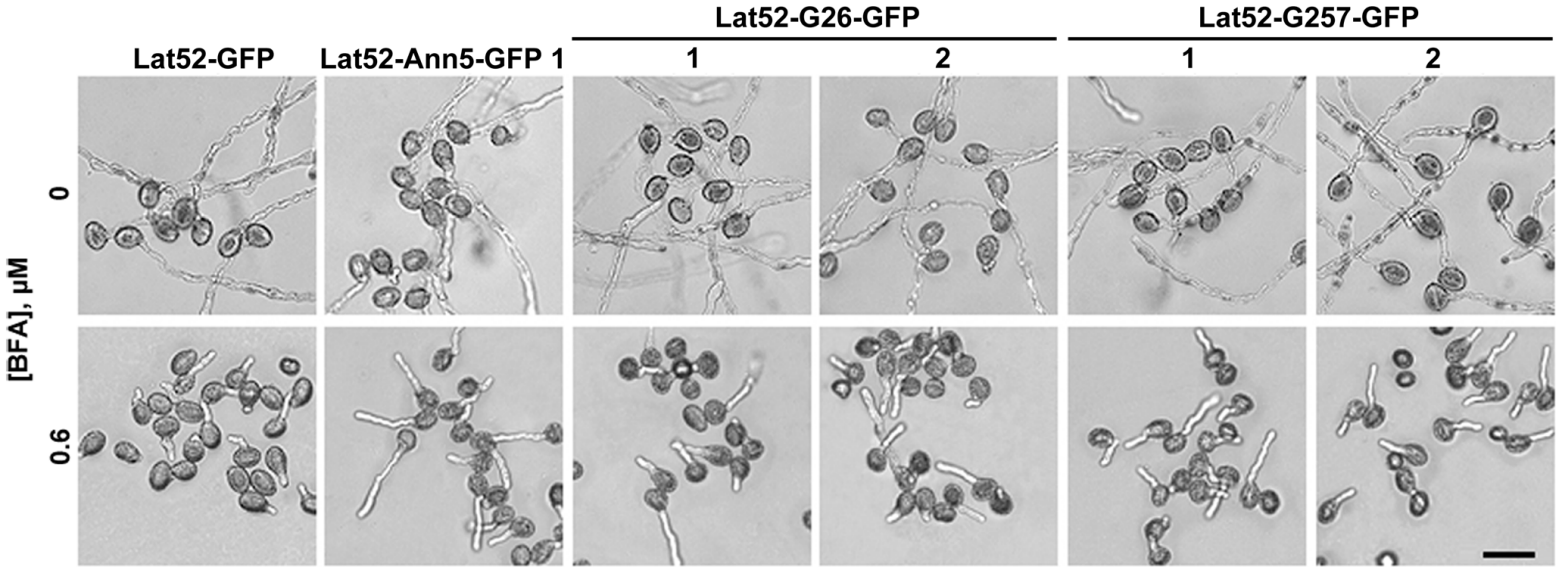
*Ann5G26EG28E* or *Ann5G257EG259E* overexpression decreased the resistance of pollen to BFA compared with *Ann5* overexpression. Pollen grains and tubes from the Lat52-GFP, Lat52-Ann5-GFP 1, Lat52-G26-GFP 1 and 2 and Lat52-G257-GFP 1 and 2 homozygous lines growing on germination medium containing 0.6 µM BFA in vitro. Medium containing 50 nM DMSO was used as the control. The images were captured after culturing for 3 h. Bar  = 50 µm.

There was no obvious pollen germination or pollen tube growth-related phenotype in lines overexpressing *Ann5G26EG28E* or *Ann5G257EG259E* under normal germination conditions. However, when 0.6 µM BFA was added, the pollen derived from Lat52-G26-GFP 1 and 2 and Lat52-G257-GFP 1 and 2 plants displayed a significant reduction in the pollen germination rate in response to BFA, in contrast to Lat52-Ann5-GFP 1 pollen, whereas all of them exhibited a greater resistance to BFA treatment than Lat52-GFP pollen (P<0.01) (Figures 7, Table 2), which suggests that Ann5 plays an important role in pollen germination and pollen tube growth via Ca^2+^-dependent membrane binding activity. In addition to the decreased resistance to BFA treatment during pollen germination compared with the Lat52-Ann5-GFP 1 line, the pollen tube growth of the Lat52-G26-GFP 1 and 2 and Lat52-G257-GFP 1 and 2 plants also exhibited a similar phenotype (Table 2). These results further confirm that *Ann5* is involved in endomembrane trafficking as a positive regulator in response to BFA treatment, and this cellular process is modulated by Ca^2+^ fluctuations that occur within pollen cells.

**Table 2:** Comparison of pollen germination rates and pollen tube lengths between the Lat52-GFP, Lat52-Ann5-GFP 1, Lat52-G26-GFP 1 and 2 and Lat52-G257-GFP 1 and 2 homozygous lines.

	Pollen germination rate, %		Average pollen tube length, µm	
BFA (µM)	0	0.6	0	0.6
Lat52-GFP	91.0±1.3	5.8±1.0	352.2±7.2	178.3±12.6
Lat52-Ann5-GFP 1	91.7±1.6	53.4±2.4**	361.7±13.3	273.4±10.3**
Lat52-G26-GFP 1	91.3±1.2	20.8±2.1**	355.8±15.9	228.7±4.6*
Lat52-G26-GFP 2	90.5±1.5	23.0±1.3**	349.1±14.6	238.5±13.6*
Lat52-G257-GFP 1	90.8±1.1	21.4±2.5**	366.5±6.9	231.2±7.4*
Lat52-G257-GFP 2	92.0±1.0	19.2±2.1**	360.3±5.1	233.9±5.4*

For pollen germination rate, the pollen germinated for 3 h in the presence of 0.6 µM BFA. Six hundred pollen grains and tubes were counted. For pollen tube length, the pollen germinated normally for 2 h and then for 2 h in the presence of 0.6 µM BFA. Three hundred pollen tubes were measured. Values represent the means ± _SD_. *P<0.05 and **P<0.01 by Student's *t* test.

### *Ann5G26EG28E* and *Ann5G257EG259E* Only Partially Rescued the Aborted Pollen Genotype in the Ann5UTR-RNAi Plant

**Figure 8:**
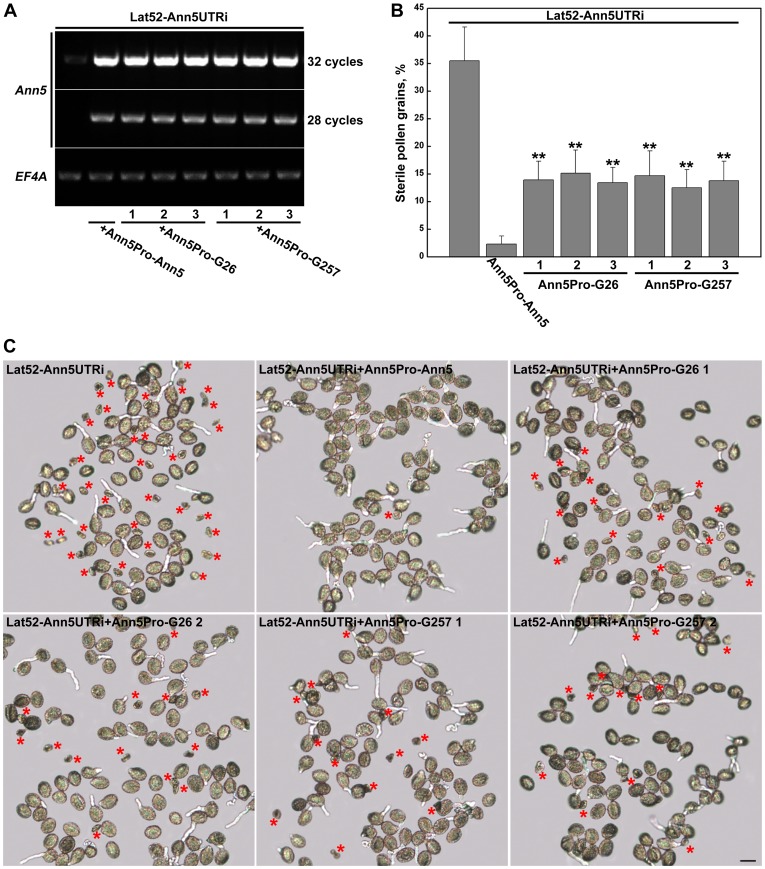
Ann5Pro-G26 and Ann5Pro-G257 only partially rescued the aborted pollen genotype in Lat52-Ann5UTRi plants. (A) The expression level of the *Ann5* transcript in Lat52-Ann5UTRi and individual complementation plants. Total RNA was isolated from the open flowers and used for RT-PCR. *EF4A* was used as the control. (B) Quantification of the sterile pollen grains from Lat52-Ann5UTRi and the individual complementation plants. Values represent the means ± _SD_. At least 500 pollen grains were counted. **P<0.01 by Student's *t* test. (C) Recovery analysis of the pollen lethality phenotype. Ann5Pro-Ann5 fully rescued the sterile pollen phenotype in the Lat52-Ann5UTRi lines. Ann5Pro-G26 and Ann5Pro-G257 only partially rescued the aborted pollen genotype. Bright-field images were obtained with a microscope 1 h after germination. Red stars indicate the aborted pollen grains. Bars  = 20 µm.

Our recent report indicated that downregulation of the function of *Ann5* in transgenic Ann5UTR-RNAi lines caused severely sterile pollen grains beginning from the bicellular stage, and the sterile phenotype could be rescued by complementation with exogenetic *Ann5* [42]. To further investigate the effect of *Ann5G26EG28E* and *Ann5G257EG259E* on pollen development, the Ann5Pro-Ann5G26EG28E or Ann5G257EG259E complementation constructs (named Ann5Pro-G26 or Ann5Pro -G257) were introduced into the Lat52-Ann5UTR-RNAi (named Lat52-Ann5UTRi) line. The Ann5Pro-Ann5 recovery line was used as the positive control. We detected the *Ann5* transcript level in the Ann5Pro-G26 and Ann5Pro-G257 complementation lines using RT-PCR. The *Ann5* transcript level in the Ann5Pro-G26 and Ann5Pro-G257 recovery lines was similar to that in the Ann5Pro-Ann5 recovery line (Figure 8A). As shown in the bright field results, the Ann5Pro-Ann5 construct was able to fully rescue the smaller and misshapen pollen phenotype in the Lat52-Ann5UTRi plant. However, the percentage of aborted pollen from the Ann5Pro-G26 or Ann5Pro-G257 recovery lines was significantly higher than that from the Ann5Pro-Ann5 complemented plant but lower than that from the Lat52-Ann5UTRi plant (Figures 8B and C). These data demonstrated that the defects observed in pollen grains of the Ann5UTR-RNAi line could be only partially recovered by Ann5 mutants at Ca^2+^-binding sites, which suggests that *Ann5* is required for pollen development via Ca^2+^-dependent membrane trafficking.

### The Velocity of Cytoplasmic Streaming in Response to BFA was Altered in Pollen Tubes Overexpressing *Ann5*

**Figure 9:**
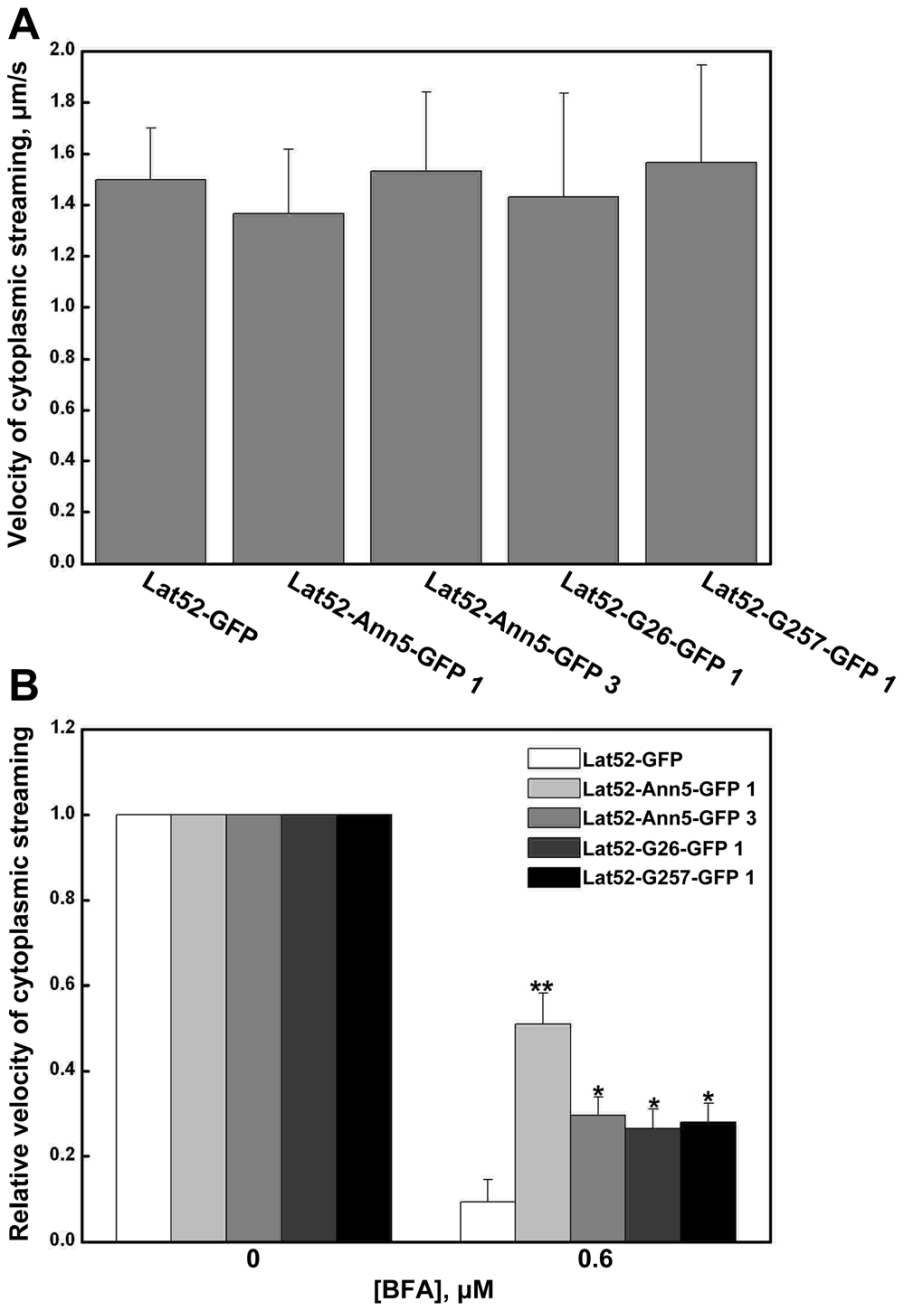
Analysis of the cytoplasmic streaming velocity in pollen tubes of the overexpression lines. (A) The velocity of cytoplasmic streaming in pollen tubes from the overexpression lines Lat52-GFP, Lat52-Ann5-GFP 1 and 3, Lat52-G26-GFP 1 and Lat52-G257-GFP 1 under normal conditions. Pollen grains were cultured on germination medium for 4 h. Cytosolic particles exhibiting continuous movement were selected at random for velocity analysis using the Image J software. Values represent the means ± _SD_ (n = 25). (B) The relative velocity of cytoplasmic streaming in pollen tubes from the Lat52-GFP, Lat52-Ann5-GFP 1 and 3, Lat52-G26-GFP 1 and Lat52-G257-GFP 1 overexpression lines in response to 0.6 µM BFA. The pollen had germinated normally for 2 h, followed by 2 h in the presence of 0.6 µM BFA. The velocity of the particles of each individual line in normal conditions was normalized to 1. The relative velocity was displayed as the proportion over the control. Values represent the means ± _SD_. *P<0.05 and **P<0.01 (n = 25) by Student's *t* test.

Our results to this point demonstrate that *Ann5* is involved in endomembrane trafficking and most likely promotes these processes in response to BFA treatment in a Ca^2+^-dependent manner. To further investigate the Ca^2+^-dependent increased resistance to BFA treatment of *Ann5*-overexpressing pollen, we observed the cytoplasmic streaming in the pollen tubes from plants overexpressing *Ann5*, *Ann5G26EG28E* or *Ann5G257EG259E*. Neither the pattern nor the velocity of the cytoplasmic streaming exhibited significant differences between the Lat52-GFP and overexpression lines (Figure 9A). This result is consistent with the fact that there was no obvious pollen germination or pollen tube growth-related phenotype in lines overexpressing *Ann5* or its mutants at Ca^2+^-binding sites under normal germination conditions. However, when the pollen tubes were treated with 0.6 µM BFA for 2 h, a significant difference was observed between the Lat52-GFP and overexpression lines. The relative velocity of the cytoplasmic streaming was reduced to a lesser degree in response to BFA in the pollen tubes overexpressing Ann5, Ann5G26EG28E or Ann5G257EG259E than that in the Lat52-GFP line. The difference in the relative velocity between the Lat52-Ann5-GFP 1 and Lat52-Ann5-GFP 3 lines was due to the distinct expression level of *Ann5* in pollen. Furthermore, the relative velocity of cytoplasmic streaming in the Lat52-Ann5-GFP 1 line was higher than that in the Lat52-G26-GFP 1 and Lat52-G257-GFP 1 lines following BFA treatment, although they had similar levels of *Ann5* expression (Figure 9B), which suggests that *Ann5* is involved in endomembrane trafficking and that it promotes cytoplasmic streaming following BFA treatment in a Ca^2+^-dependent manner.

## Discussion

In plant cells, annexins represent approximately 0.1% of the total protein. Despite many years of investigation since the identification of the first plant annexin from tomato [50], the main physiological significance of plant annexins remains largely unknown, and most of our knowledge about plant annexins is focused on their structure and in vitro protein function. *Ann5* is expressed abundantly in mature pollen grains and elongating pollen tubes. Furthermore, downregulation of *Ann5* leads to a sterile pollen grain phenotype [42]. Here, we provide both biochemical and physiological evidence to interpret the molecular mechanisms underlying the function of *Ann5* in plant pollen development and pollen tube growth.

### Ann5 Binds to Phospholipid Membranes and Actin Filaments in Vitro

Both animal and plant annexins are able to bind Ca^2+^ and reversibly interact with negatively charged phospholipids at the periphery of membranes containing acidic phospholipids in a calcium-dependent manner [39], [51]–[54]. Annexins have further been shown to participate in many in vivo cellular processes [55], [56]. In this work, we focused on annexin5 in *Arabidopsis*, which is predominantly expressed in pollen [42]. Recombinant Ann5 efficiently binds to liposomes containing a 1∶1 mixture of PC and PS in a Ca^2+^-dependent manner at neutral pH (Figure 1C and D). The calcium concentration required for Ann5 phospholipid binding was in the low-micromolar range (approximately 0.5 µM), and thus, binding is most likely modulated by Ca^2+^ fluctuations that occur within plant cells.

Using a structural mimetic of homology modeling, the conserved endonexin fold (GXGTD-(37 residues)-E/D) is responsible for binding Ca^2+^ and is present in only the AB and DE loops of the first or fourth repeats in *Arabidopsis* annexin5 (Figures 1A and 1B). To identify key residues for membrane binding and further confirm that Ca^2+^ regulates the association between Ann5 and phospholipid membranes, we characterized its Ca^2+^-dependent phospholipid binding in detail using two mutant proteins Ann5G26EG28E and Ann5G257EG259E, in which the conserved glycine residues in the endonexin sequences GXGTD-(37 residues)-E/D of repeats I and IV were replaced by glutamic acid residues. As expected, these two mutant proteins exhibited significant decreases in phospholipid binding in the presence of various concentrations of Ca^2+^ when compared with Ann5 (Figures 1C and D). The reduction of the mutants binding to phospholipids presents two possibilities. First, the glycine residue in the endonexin motif of Ann5 may be the critical part of the phospholipid-binding sites and other amino acids residues may be involved in assembly of the intact site. Second, the crucial glycine residue with other amino acids may stabilize the conformation of the binding between Ann5 and phospholipids. Similarly, recombinant AnnAt1 and AnnAt4 with amino acid changes in the conserved acidic residues (D/E) of the Ca^2+^-binding motifs exhibited reduced Ca^2+^-binding activity and negatively affected AnnAt-binding activity [34], implying that the GXGTD-(37 residues)-E/D motifs were pivotal for calcium binding by plant annexins. In addition, the quadruple mutant, Ann5G26EG28EG257EG259E, could not completely abolish the Ca^2+^-dependent phospholipid-binding activity of Ann5, suggesting that other amino acids of endonexin fold, such as E/D, were also crucial for calcium binding of Ann5.

Interestingly, small amounts of Ann5 and its two mutants were confirmed to associate with phospholipids at neutral pH, even in the absence of calcium (Figure 1C), which was different from the behavior of vertebrate annexins. This calcium-independent membrane binding has also been reported in other plant homologues. For example, 20% of AnxGh1 and AnxCa32 from bell pepper (*Capsicum annuum*) and cotton (*Gossypium hirsutum*), respectively, bound to lipid vesicles in the absence of Ca^2+^ at neutral pH [53]. The two different binding modes for Ann5 are most likely interconnected because annexin-membrane association has been demonstrated to be highly sequential and complicated and show consonance with respect to calcium. Ca^2+^-independent membrane binding likely serves as a platform for the binding of an annexin population whose membrane binding is Ca^2+^ dependent, suggesting that there can be resident annexin population at membranes ready to sequester Ca^2+^ and perhaps undergo a change of function as a consequence [37], [53], [57], [58]. The Ca^2+^-independent phospholipid-binding Ann5 may represent a proportion of the population undergoing membrane insertion. Some plant annexins, such as wheat p39 and p22.5, AnnAt1, are known to exist in the fully or partially membrane-inserted form [79]–[81]. Additionally, Hofmann et al. (2002) found that another unique feature of plant annexins is their tendency to form calcium-independent oligomers [82]. Such a structure would be facilitate the interaction between Ann5 and membranes in low calcium ion concentrations and be suitable for the complex physical processes in pollen cells.

Several vertebrate annexins, such as Annexin A2, which is required for the actin-dependent, apical transport of raft-associated sucrase-isomaltase-carrying vesicles in polarized epithelial cells, participate in actin remodeling [59], [60]. Although many plant annexins possess the IRI motif, which is necessary for F-actin binding to myosin, only a few have been found to bind filamentous actin, and this interaction appears to be species specific [38]. For example, *Mimosa* annexin induced F-actin to form thick bundles in the presence of Ca^2+^ in vitro and was shown to be involved in pulvinar nyctinastic movements [61]. Annexins p34 and p35 from tomato were found to bind to F-actin, but not globular actin, and this interaction was calcium- and pH-dependent [52]. Two annexins from zucchini associated with the membrane and bound to zucchini-derived F-actin [62]. Based on the results of high-speed co-sedimentation assays, the recombinant Ann5 studied here bound to F-actin in vitro (Figures 4A and B). Furthermore, the Ann5 sequence contained a partially conserved F-actin-binding motif, IQI, in repeat III. However, the precise role and functional significance of the annexin5–actin interaction was difficult to establish in this study because the pollen grains and tubes in the *Ann5*-overexpressing lines displayed a phenotype similar to that of wild-type when Lat B was used to destroy the F-actin organization in the pollen cell (Figures 5A to C). Actin filaments may serve as molecular tracks for Ann5 in endocytosis, vesicle transport and motility.

### Ann5 Promotes Membrane Trafficking and Cytoplasmic Streaming in Vivo

Pollen germination and tube growth require continued fusion with the plasma membrane by trafficking vesicles originating from the Golgi apparatus. To investigate the function of *Ann5* in pollen cellular processes, we treated pollen cells in *Ann5*-overexpressing lines with BFA, an inhibitor of secretion and vacuolar protein transport. BFA not only promotes a rapid release of COPI coat proteins from the Golgi apparatus into the cytosol and blocks ER to Golgi transport but also affects trafficking in the endocytic pathway resulting from disruption of the Golgi apparatus [45]. Thus, BFA interferes with endomembrane trafficking and strongly inhibits pollen germination and pollen tube growth in a dose-dependent manner [46], [47]. For example, BFA obstructs the secretion of cell wall material and leads to growth arrest in tobacco pollen tubes [63]. Moreover, the inhibited secretion and enhanced endocytosis that are induced by BFA are responsible for the retarded growth of pollen tubes of *Picea meyeri* [46]. On the other hand, BFA possesses some secondary effects that inhibit membrane traffic in the secretory and endocytic pathways. As has been previously reported [64], [65], BFA can dissipate the typical, oscillating tip-focused calcium gradient that is normally associated with pollen cell growth.

In this study, we demonstrated that both pollen germination and pollen tube growth of *Ann5*-overexpressing plants significantly increased their resistance to BFA treatment compared with the control line (Figure 3, Table 1). Vesicles shuttle among the endoplasmic reticulum, Golgi apparatus, plasma membrane and endosomes in plant cells. In general, there are two steps for a pollen cell to transport its cargo. First, trafficking vesicles arrive at the target membrane along microfilaments that provide tracks for vesicle movement. Then, membrane fusion of the two apposing bilayers is accomplished by a special set of proteins called SNAREs, and the two previously separate organelles now have a direct community [36], [51]. We focused on the involvement of *Ann5* in endomembrane trafficking. Ann5 likely promotes vesicle transport following BFA stimulation by binding to negatively charged phospholipids in a Ca^2+^-dependent manner and to actin filaments that served as molecular tracks in pollen grains and tubes. Annexin A2 is involved in the biogenesis of multivesicular transport intermediates that are destined for late endosomes in mammals [51], [66]. In *Arabidopsis*, AnnAt3, 4, 5 and 8 are more closely related to human annexins based on phylogenetic analysis. RNA interference–mediated knockdown of the annexin *AnnAt3* caused the trans-Golgi network (TGN) and multivesicular body (MVB) markers to colocalize and block vacuolar transport in tobacco mesophyll protoplasts. It has been shown that AnnAt3 is required for the final step of MVBs as a transport carrier from the TGN to the vacuole [67]. The *Ann5*-overexpressing pollen cell exhibited a higher velocity for cytoplasmic streaming and vesicle transport than control pollen cells following BFA treatment, further suggesting that Ann5 promoted endomembrane trafficking (Figures 9A and B). Cytoplasmic streaming is fundamentally under the control of myosin motors moving along actin filament bundles that are involved in the intracellular transport of organelles and vesicles [51], [68], [69]. In addition to the fact that actin filaments serve as molecular tracks for Ann5 in vesicle trafficking, Ann5 can bind to F-actin in vitro and may be involved in the dynamic rearrangement of actin through an unknown mechanism (Figures 4A and B). Alterations in the arrangement of actin cables greatly influence the pattern and velocity of cytoplasmic streaming. Ann5 binds to actin and associates with phospholipid membranes in a Ca^2+^-dependent manner (Figures 1A to D, Figures 4A and B), making it an ideal regulator of endomembrane trafficking where the cell membrane and cytoskeleton interact and are modulated by Ca^2+^ within pollen cells.

It has been well established that the growing pollen tube possesses a “tip-focused“ gradient of free calcium, in which the cytosolic concentration of free Ca^2+^ extends from 2–10 µM (in the apical region) to 20–200 nM (in the shank region) [70]–[72]. The growth rates of pollen tubes and the cytosolic Ca^2+^ concentration also synchronously oscillate with the same period and phase [14], [73], [74]. Moreover, Ca^2+^ has been reported to affect organelle and vesicle motility along actin filaments by inactivating the myosin motor through binding to its calmodulin light chain [75]. In addition to the results showing that the Ann5G26EG28E and Ann5G257EG259E proteins exhibited significantly less phospholipid binding compared with Ann5 in vitro (Figures 1C and D), the pollen germination, pollen tube growth and velocity of cytoplasmic streaming in *Ann5G26EG28E-* and *Ann5G257EG259E-*overexpressing plants were less resistant to BFA treatment than those in *Ann5*-overexpressing plants (Figure 7, Table 2, Figures 9A and B). These results further confirmed that Ann5 promoted endomembrane trafficking in response to BFA treatment and that this cellular process was modulated by Ca^2+^ fluctuations occurring within pollen cells. In addition, the abortion of pollen grains in the Ann5UTR-RNAi lines could be only partially recovered by *Ann5G26EG28E* and *Ann5G257EG259E*, while *Ann5* at a similar transcript level could fully rescue the sterile phenotype (Figures 8A to C). Although Ann5, Ann5G26EG28E and Ann5G257EG259E were diffusely distributed throughout the pollen tube (Figure 6E) [42], the quantitative fluctuation and polar distribution of Ca^2+^ determined the differences in the phospholipid membrane binding activity of Ann5 in distinct spatial positions and developmental phases of pollen cells. Tight regulation of the endomembrane trafficking of Ann5 in pollen involved Ca^2+^ as a second messenger. Ann5 might act as a Ca^2+^ sensor and thus play an important role in controlling the processes of pollen development, germination and tube growth.

In summary, our results clearly demonstrated that pollen germination, pollen tube growth and cytoplasmic streaming are more resistant to BFA treatment in a Ca^2+^-dependent manner in *Ann5*-overexpressing plants. Considering the results that Ann5 binds to membranes, Ca^2+^ and actin filaments in vitro, we suggest that Ann5 exerts a major influence on the physiological processes involved in pollen development and growth by modulating membrane trafficking in a Ca^2+^-dependent manner.

## Materials and Methods

### Plant Materials, Growth Conditions and Transformation

*Arabidopsis thaliana* ecotype Columbia-0 (Col-0) was used as the background for all of the wild-type and overexpression lines described in this study. *Arabidopsis* seeds were stratified for 3 days at 4°C and then sterilized in 0.5% sodium hypochlorite for 10 min and plated on 0.5×Murashige and Skoog (MS) agar medium supplemented with 2% sucrose (pH 5.8). After 10 days, seedlings were transferred to soil and then grown under long-day conditions (16-h light/8-h dark photoperiod) and moderate humidity. The resulting binary plasmid was introduced into *Agrobacterium tumefaciens* strain GV3101 and used to transform *Arabidopsis* plants using a standard floral dipping method [76]. The harvested seeds were selected on an MS medium with 50 mg/L hygromycin or 35 mg/L kanamycin (Roche). Eventually, homozygotes were identified by fluorescence observation of pollen grains and selected for further analysis.

### RT-PCR and Vector Construction

Wild-type *Arabidopsis* total RNA was isolated from open flowers using a Total RNA Isolation Kit (TIANGEN). The cDNA was produced by reverse transcription with MMLV reverse transcriptase (TaKaRa Bio) in accordance with the manufacturer's recommendations. The full-length cDNA sequence of *Ann5* was amplified from wild-type flower cDNA and then cloned into the pCAMBIA1300-Lat52-GFP vector at the XbaI/BamHI sites to generate the pCAMBIA1300-Lat52-Ann5-GFP construct. PCR-based, site-directed mutagenesis (TaKaRa Bio) was performed to produce the mutants *Ann5G26EG28E* and *Ann5G257EG259E* using *Ann5* cDNA as a template. Primer pairs were synthesized such that the conserved glycine residues were replaced by glutamic acid residues. The XbaI and BamHI fragments of *Ann5G26EG28E* or *Ann5G257EG259E* were cloned into the pCAMBIA1300-Lat52-GFP vector to generate the pCAMBIA1300-Lat52-Ann5G26EG28E/Ann5G257EG259E-GFP vectors. A 1.8-kb region of the upstream noncoding region of *Ann5* (*Ann5* promoter) was isolated from wild-type *Arabidopsis* genomic DNA and then cloned into the pBI121 vector to generate the pBI121-Ann5Pro construct. The XbaI and SacI fragments of *Ann5*, *Ann5G26EG28E* and *Ann5G257EG259E* were cloned into PBI121-Ann5Pro, and the resulting complementation constructs PBI121-Ann5Pro-Ann5, PBI121-Ann5Pro-Ann5G26EG28E and PBI121-Ann5Pro-Ann5G257EG259E were introduced into the 1300-Lat52-Ann5UTR-RNAi line as before [42]. The cDNA of *Ann5*, *Ann5G26EG28E*, *Ann5G257EG259E* and *Ann5G26EG28EG257EG259E* was subcloned into the pGEX-4T1 (Amersham) and PET30a (Novagen) vectors at the BamHI/SalI restriction sites for expression in *E. coli* strain BL21. Prime STAR HS DNA polymerase (TaKaRa Bio) was used for all PCR reactions. The amplified sequences were A-tailed and cloned into the pMD19-T vector (TaKaRa Bio). All amplified fragments were verified through DNA sequencing analyses at BGI (Shenzhen, China). The primers used for cloning are described in Table S1.

To confirm the expression level of *Ann5* in various tissues and lines, RT-PCR was performed to amplify the cDNA fragments of *Ann5* using specific primers. The housekeeping gene *EF4A* was used as an internal positive control with specific primers to amplify an approximately 0.5-kb DNA fragment. PCR products were verified by 1% agarose gel electrophoresis.

### Pollen Germination Assay and Characterization of Pollen Grains and Tubes in Vitro

For pollen germination and pollen tube growth in vitro, pollen was harvested from open flowers and placed onto *Arabidopsis* pollen germination medium containing 0.36 mg/mL CaCl_2_, 0.01 mg/mL myo-inositol, 1% (w/v) gelatin, 0.08 mg/mL H_3_BO_3_ and 20% (w/v) sucrose and solidified with 1% (w/v) agarose LMP. The plates were cultured at 22°C under moist conditions in the dark. A Leica DFC4200C microscope equipped with a 10× objective was used to observe pollen grains and pollen tubes. The average pollen tube length was calculated using the Image J software (http://rsbweb.nih.gov/ij/; version 1.38), and pollen germination rates were calculated by dividing the total number of germinated tubes by the number of grains. At least three experiments were conducted for each condition. To determine the effect of BFA (Sigma-Aldrich) on pollen germination, pollen from representative lines was incubated in *Arabidopsis* pollen germination medium containing 0.3 µM or 0.6 µM BFA for 3 h in vitro. Because BFA was dissolved in dimethyl sulfoxide (DMSO), DMSO was added as a control. To examine the effect of BFA on pollen tube growth, pollen grains were incubated in standard germination medium for 2 h and then treated with 0.3 µM or 0.6 µM BFA. Pollen tube length was measured after culturing for 2 h. To determine the effect of LatB (Invitrogen) on pollen germination and tube growth, various concentrations of LatB were added to the germination medium, as described for the BFA treatment.

### Subcellular Localization Using Confocal Laser Scanning Microscopy

The pollen tubes of the homozygous pCAMBIA1300-Lat52-GFP, pCAMBIA1300-Lat52-Ann5G26EG28E-GFP and pCAMBIA1300-Lat52-Ann5G257EG259E-GFP lines were observed using an Olympus FV100 confocal microscope with a 100× oil objective. GFP was excited using a 488-nm argon laser, and emission was detected through 525±5.5 nm filters. Serial confocal optical sections were taken at a step size of 0.5 µm, and two Kalman-filtered scans were averaged for each optical section.

### Protein Purification and F-Actin Binding Assay

To investigate the biochemical basis for the function of Ann5, we produced recombinant Ann5 as an N-terminal glutathione S-transferase (GST) fusion. Fusion proteins were expressed in the *E. coli* BL21 DE3 strain by induction with 0.5 mM isopropyl β-D-thiogalactopyranoside (IPTG) overnight at 22°C. Cells were collected by centrifugation and resuspended in PBS (137 mM NaCl, 2.7 mM KCl, 10 mM Na_2_HPO_4_ and 2 mM KH_2_PO_4_, pH 8.0). GST-Ann5 was affinity purified using a glutathione Sepharose resin according to the manufacturer's recommended protocol (Bio-Rad). The purified protein was dialyzed overnight against buffer A3 (2 mM Tris-HCl, 0.2 mM CaCl_2_, 0.2 mM ATP and 0.2 mM DTT, pH 8.0).

A high-speed co-sedimentation assay was performed to determine the binding activity of the GST-Ann5 recombinant protein to F-actin, as previously described [5]. Equal amounts of the supernatant and pellet were separated by 12% SDS-PAGE and stained with Coomassie Brilliant Blue R 250. The intensities of the resulting bands were quantified by densitometry using the Image J software.

### Phospholipid Vesicle Binding Assay

To determine the effect of Ca^2+^ on Ann5 and phospholipid membrane binding, we assessed the binding activity of the recombinant protein to liposomes composed of PC/PS (1∶1) using co-sedimentation experiments, as previously described, with slight modifications [15]. The recombinant His6-Ann5, His6-Ann5G26EG28E, His6-Ann5G257EG259E and His6-Ann5G26EG28EG257EG259E proteins were purified by nickel-nitrilotriacetic acid (Ni-NTA) His-binding resin following the manufacturer's protocol (QIAGEN). The purified proteins were dialyzed overnight against phospholipid binding buffer (30 mM HEPES, pH 7.5, 50 mM KCl). Fifty microliters each of 20 mg/mL phosphatidylserine (PS; Sigma-Aldrich) and phosphatidylcholine (PC; Sigma-Aldrich) (dissolved in chloroform) were shelled by N_2_ gas, and the resulting lipid residue was resuspended in 1 mL of phospholipid-binding buffer and sonicated for 20 min to prepare phospholipid vesicles. For each protein, a 50-µg protein solution was incubated with liposomes (1∶1 PC/PS) in the presence of increasing Ca^2+^ concentrations (0, 0.2, 0.5, 1, 10,50,100 and 200 µM) at neutral pH for 20 min at room temperature. Then, the reaction mixtures were centrifuged at 20,000 g, and the protein bound to the phospholipid vesicles in the pellet was separated by SDS-PAGE and stained with Coomassie Brilliant Blue R. The densitometric analysis of the Ann5 band was performed using the Image J software.

### Analysis of Cytoplasmic Streaming

The cytoplasmic streaming of pollen tubes was observed using a Leica DFC4200C microscope equipped with a 100× oil objective. Images were captured at 0.5-s intervals over 5 min. Pollen grains were cultured on *Arabidopsis* pollen germination medium for 4 h under normal conditions. To investigate the effect of BFA on cytoplasmic streaming, pollen was germinated normally for 2 h and then treated with 0.6 µM BFA for 2 h. The cytosolic particles exhibiting continuous movement were selected at random for velocity analysis using the Image J software.

### Sequence Alignment and Structure Modeling

Multiple sequence alignment of the deduced amino acid sequences of repeats I and IV from *Arabidopsis* AnnAt1 to 8 was performed using DNAMAN (Lynnon Biosoft). A homology model of Ann5 was produced by the Swiss-Model program (http://www.expasy.ch/) and then colored using PyMOL software. The model was based on template 2zocA (2.00 A), and the QMEAN Z-Score was −1.018.

## Supporting Information

Figure S1 **Expression and purification of recombinant His6-Ann5, His6-Ann5G26EG28E, His6-Ann5G257EG259E and His6-Ann5G26EG28EG257EG259E.** (A) and (B) The lateral (A) and top (B) faces of the predicted three-dimensional structure of the Ann5 protein in Figure 1 (B). (C) Ann5 and its mutants fused to a His-tag were expressed in the *Escherichia coli* BL21 (DE3) strain and purified to homogeneity by affinity chromatography. Proteins were separated by SDS-PAGE and stained with Coomassie Brilliant Blue R. Lane 1, crude extract from bacterial cells without isopropylthio-β-galactoside (IPTG) induction; Lanes 2, 3 and 4, crude extracts of His6-Ann5, His6-Ann5G26EG28E and His6-Ann5G257EG259E, respectively, from bacterial cells with 0.5 mM IPTG induction; Lanes 5, 6 and 7, purified recombinant His6-Ann5, His6-Ann5G26EG28E and His6-Ann5G257EG259E, respectively. (D) Equal amounts of recombinant His6-Ann5, His6-Ann5G26EG28E, His6-Ann5G257EG259E and His6-Ann5G26EG28EG257EG259E (50 µg) were separated by SDS-PAGE and stained with Coomassie Brilliant Blue R. (E) Phospholipid-binding properties of the recombinant GST protein. GST protein was incubated with liposomes (1∶1 PC/PS) in the presence of increasing Ca^2+^ concentrations, as indicated, at neutral pH. (-) denotes that the reaction mixtures contained neither liposomes nor Ca^2+^. The results are representative of three independent experiments. (F) Phospholipid-binding properties of the recombinant His6-Ann1 protein. His6-Ann1 protein was incubated with liposomes (1∶1 PC/PS) in the presence of increasing Ca^2+^ concentrations, as indicated, at neutral pH. The results are representative of three independent experiments.
(TIF)

Figure S2 **Binding activity of GST and GST-CROLIN1 to actin filaments as revealed by a high-speed co-sedimentation assay.** A high-speed co-sedimentation assay was performed to assess the ability of GST and CROLIN1 to bind to F-actin. Either 2 µM GST or 1 µM CROLIN1 was incubated with 4 µM F-actin at 22°C for 1 h and then centrifuged at 100,000 g for 1 h. Equal amounts of the supernatant (S) and pellet (P) were separated by SDS-PAGE and stained with Coomassie Brilliant Blue R. When alone, most GST or CROLIN1 was found in the supernatant as a soluble protein. CROLIN1 accumulation in the pellet indicates F-actin binding. GST could not interact with F-actin. At least three similar results were obtained, and a representative is shown.
(TIF)

Table S1 **Primer information of** ***Ann5*** **.**
(TIF)
