# Supplementary figures and images for: Annexin5 Plays a Vital Role in Arabidopsis Pollen Development via Ca2+-Dependent Membrane Trafficking
Source: PLoS One. 2014 Jul 14;9(7):e102407. doi: 10.1371/journal.pone.0102407 (PMC4097066; doi:10.1371/journal.pone.0102407)

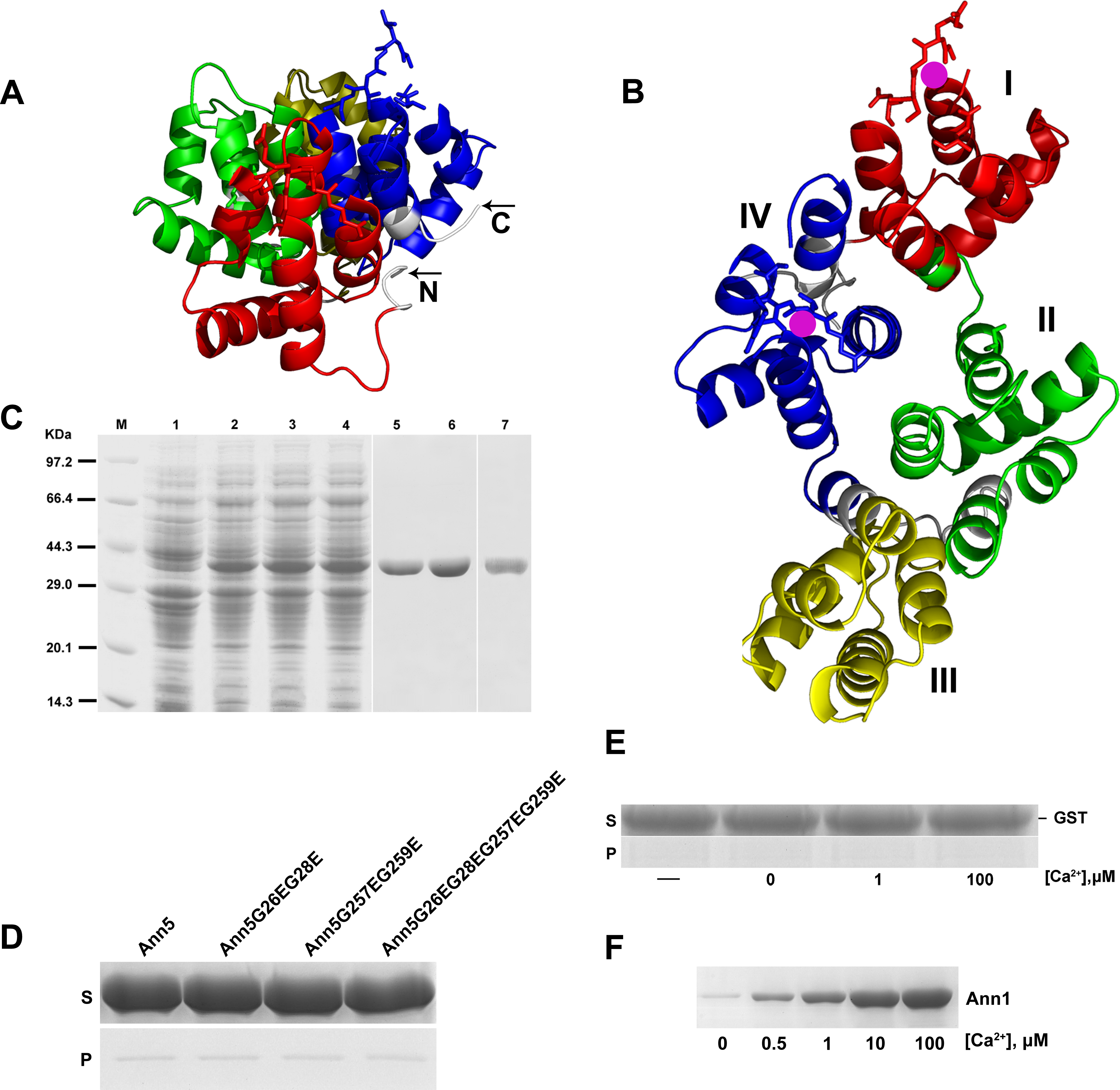

Supplement: Figure S1 — Expression and purification of recombinant His6-Ann5, His6-Ann5G26EG28E, His6-Ann5G257EG259E and His6-Ann5G26EG28EG257EG259E. (A) and (B) The lateral (A) and top (B) faces of the predicted three-dimensional structure of the Ann5 protein in Figure 1 (B). (C) Ann5 and its mutants fused to a His-tag were expressed in the Escherichia coli BL21 (DE3) strain and purified to homogeneity by affinity chromatography. Proteins were separated by SDS-PAGE and stained with Coomassie Brilliant Blue R. Lane 1, crude extract from bacterial cells without isopropylthio-β-galactoside (IPTG) induction; Lanes 2, 3 and 4, crude extracts of His6-Ann5, His6-Ann5G26EG28E and His6-Ann5G257EG259E, respectively, from bacterial cells with 0.5 mM IPTG induction; Lanes 5, 6 and 7, purified recombinant His6-Ann5, His6-Ann5G26EG28E and His6-Ann5G257EG259E, respectively. (D) Equal amounts of recombinant His6-Ann5, His6-Ann5G26EG28E, His6-Ann5G257EG259E and His6-Ann5G26EG28EG257EG259E (50 µg) were separated by SDS-PAGE and stained with Coomassie Brilliant Blue R. (E) Phospholipid-binding properties of the recombinant GST protein. GST protein was incubated with liposomes (1∶1 PC/PS) in the presence of increasing Ca2+ concentrations, as indicated, at neutral pH. (-) denotes that the reaction mixtures contained neither liposomes nor Ca2+. The results are representative of three independent experiments. (F) Phospholipid-binding properties of the recombinant His6-Ann1 protein. His6-Ann1 protein was incubated with liposomes (1∶1 PC/PS) in the presence of increasing Ca2+ concentrations, as indicated, at neutral pH. The results are representative of three independent experiments. (TIF) [file pone.0102407.s001.tif]

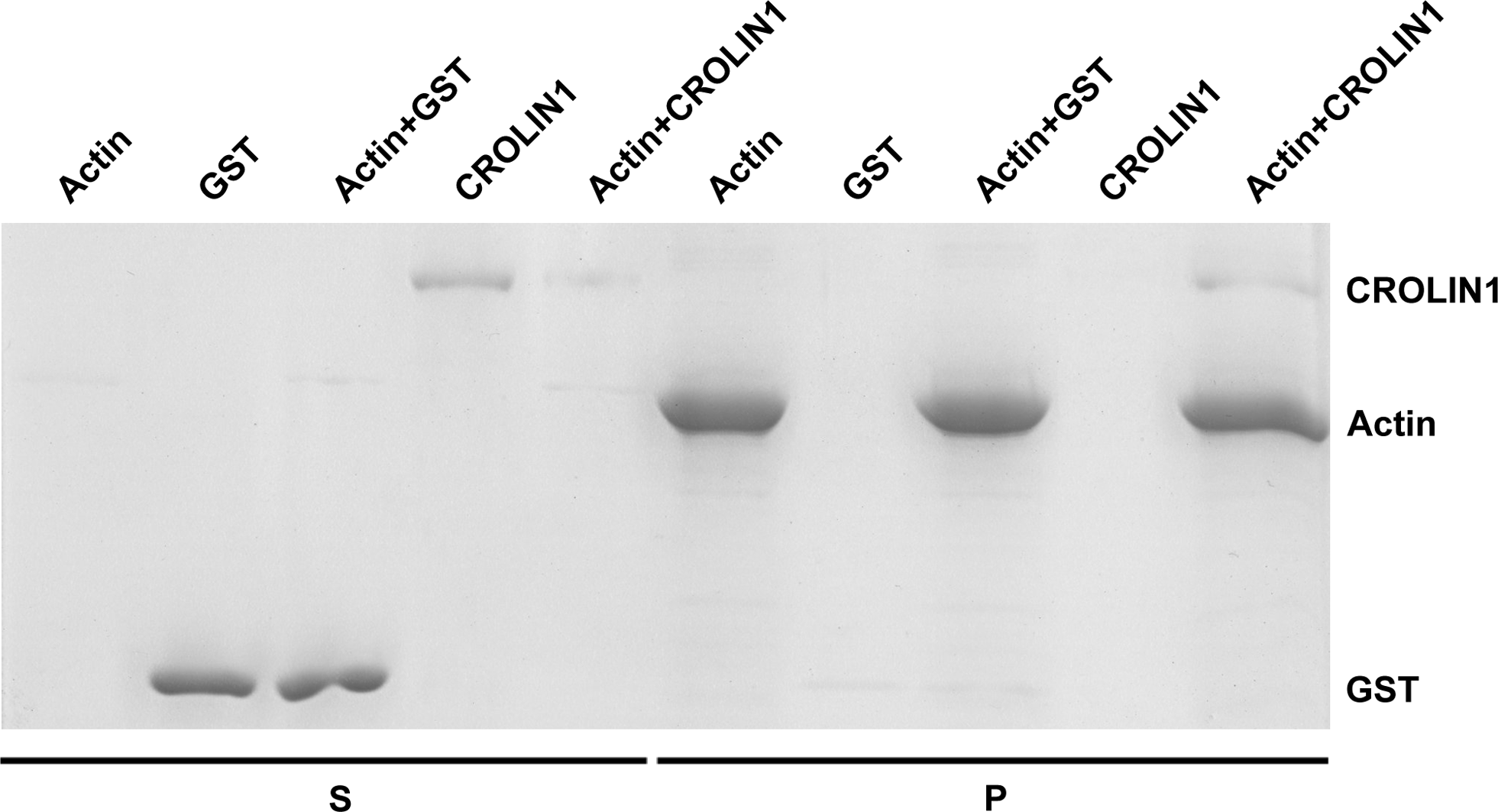

Supplement: Figure S2 — Binding activity of GST and GST-CROLIN1 to actin filaments as revealed by a high-speed co-sedimentation assay. A high-speed co-sedimentation assay was performed to assess the ability of GST and CROLIN1 to bind to F-actin. Either 2 µM GST or 1 µM CROLIN1 was incubated with 4 µM F-actin at 22°C for 1 h and then centrifuged at 100,000 g for 1 h. Equal amounts of the supernatant (S) and pellet (P) were separated by SDS-PAGE and stained with Coomassie Brilliant Blue R. When alone, most GST or CROLIN1 was found in the supernatant as a soluble protein. CROLIN1 accumulation in the pellet indicates F-actin binding. GST could not interact with F-actin. At least three similar results were obtained, and a representative is shown. (TIF) [file pone.0102407.s002.tif]

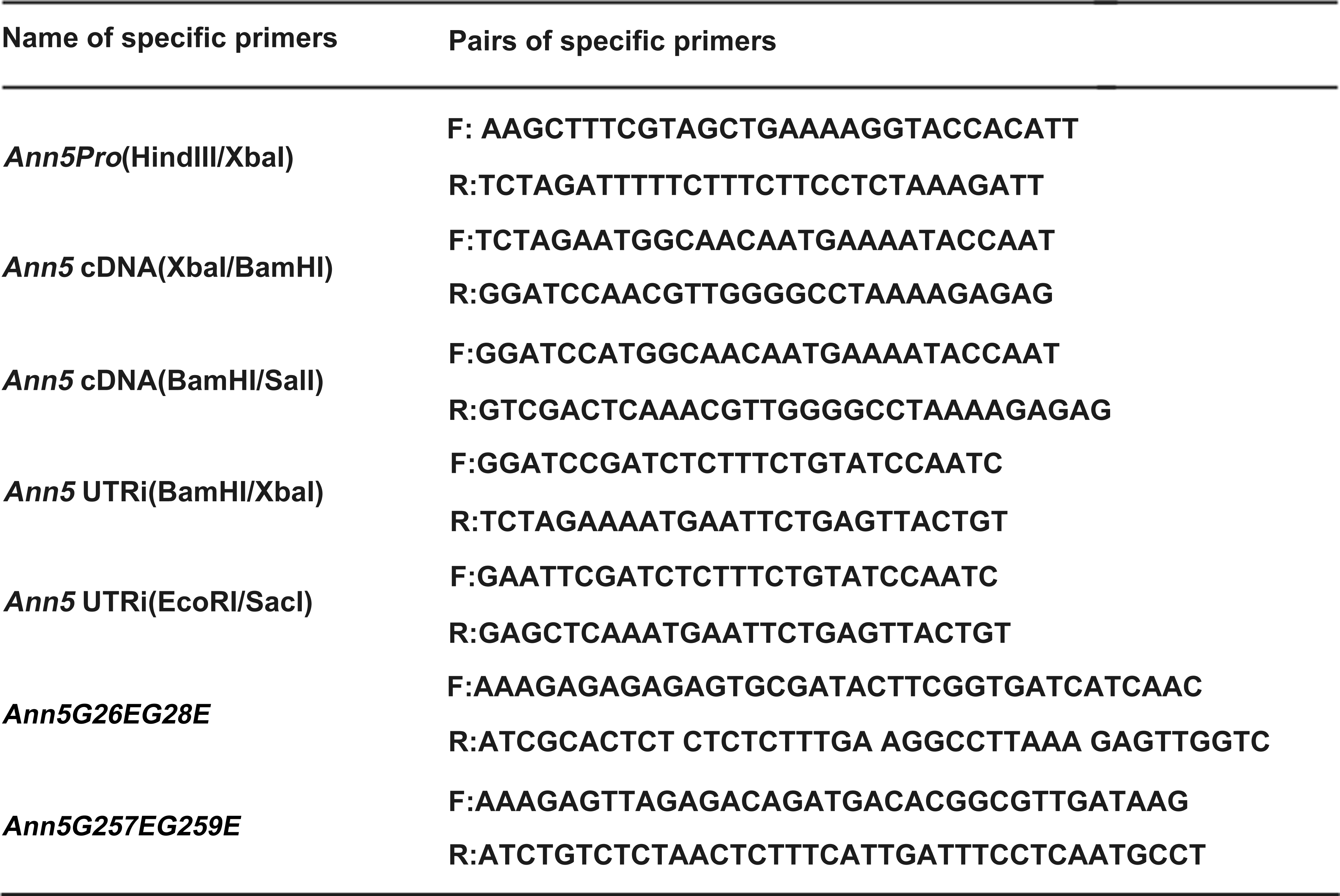

Supplement: Table S1 — Primer information of Ann5 . (TIF) [file pone.0102407.s003.tif]
